# Engineering Nanostructural Routes for Enhancing Thermoelectric Performance: Bulk to Nanoscale

**DOI:** 10.3389/fchem.2015.00063

**Published:** 2015-11-10

**Authors:** Rajeshkumar Mohanraman, Tian-Wey Lan, Te-Chih Hsiung, Dedi Amada, Ping-Chung Lee, Min-Nan Ou, Yang-Yuan Chen

**Affiliations:** ^1^Department of Engineering and System Science, National Tsing Hua UniversityHsinchu, Taiwan; ^2^Institute of Physics, Academia SinicaTaipei, Taiwan; ^3^Nano Science and Technology, Taiwan International Graduate Program, Institute of Physics, Academia SinicaTaipei, Taiwan; ^4^Department of Physics, National Taiwan UniversityTaipei, Taiwan; ^5^Research Center for Electronics and Telecommunication, Indonesian Institutes of SciencesBadung, Indonesia

**Keywords:** thermoelectrics, nanowires, AgSbTe_2_, doping, nanoprecipitate, Bi_2_Te_3_, PbTe, CoSb_3_

## Abstract

Thermoelectricity is a very important phenomenon, especially its significance in heat-electricity conversion. If thermoelectric devices can be effectively applied to the recovery of the renewable energies, such as waste heat and solar energy, the energy shortage, and global warming issues may be greatly relieved. This review focusses recent developments on the thermoelectric performance of a low-dimensional material, bulk nanostructured materials, conventional bulk materials etc. Particular emphasis is given on, how the nanostructure in nanostructured composites, confinement effects in one-dimensional nanowires and doping effects in conventional bulk composites plays an important role in ZT enhancement.

## Introduction

Thermoelectric (TE) materials can directly contribute toward a global joint solution because they are capable of converting a thermal gradient into a voltage, and vice-versa, and thus to recover waste heat (Bell, 2008). Many advantages of this technology can be cited with respect to other approaches to refrigeration or power generation: Compactness and quietness, scalability, no moving parts, long, and reliable working life, local cooling or power generation, no need of maintenance and remarkably, the abundance of waste heat sources present in household and industrial processes (Rowe, 2006).

The performance of TE devices is assessed using the dimensionless figure of merit *ZT* = α^2^σT/*κ*, where α, σ, T, and *κ* are the Seebeck coefficient, the electrical conductivity, the absolute temperature, and the thermal conductivity, respectively. Because α, σ, and the electronic contribution to *κ* involve band structures (e.g., energy gap *E*_*g*_, effective mass carrier m^*^), and scattering mechanisms, controlling the parameters independently is difficult (Bell, 2008). Therefore, a *ZT*-value of 1 has long been considered a benchmark for practical TE materials. Based on the above relationship, optimally performing TE materials should possess high electrical conductivity, a large Seebeck coefficient, and low thermal conductivity (Bell, 2008). This review covers the latest advancement in TE technology focusing on the nanostructural approaches, provides comprehensive review on recent developments in nanowires and also highlights some of the most promising thermoelectric material system including Bi-Te alloys, CoSb_3_ skutterudites, PbTe, AgSbTe_2_ etc.

## Research progress on nanostructured TE materials

### One-dimensional TE materials: Nanowires

Low-dimensional TE materials such as quantum wells and nanowires are supposed to have excellent thermoelectric properties than their bulk counterparts, because of increase in the density of states (DOS) near Fermi level by quantum confinement which tends to enhance the Seebeck coefficient. And also effectively scatters phonon over a large mean free path (mfp) by high density of interfaces, hence resulting in the lower lattice thermal conductivity. Remarkable enhancement of *ZT* has been reported in one- dimensional (1D) thermoelectric materials.

**Figure 1:**
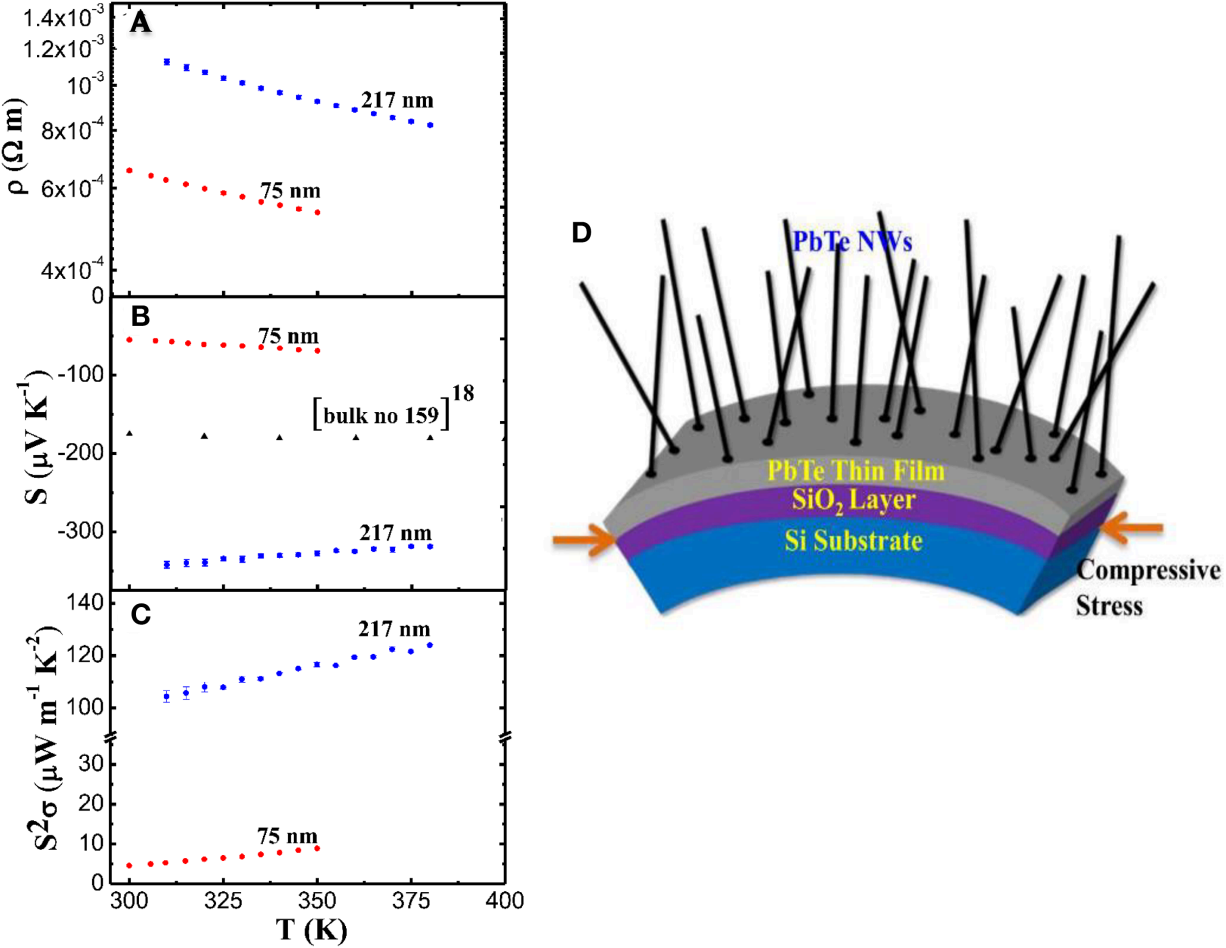
**(A)** Resistivity measurement; **(B)** Seebeck coefficient measurement; **(C)** power factor for PbTe NWs with dw = 75 and 217 nm; and **(D)** A representation of the growth mechanism in PbTe NWs using the catalyst-free stress-induced method. Reproduced from Dedi et al. (2013).

**Figure 2:**
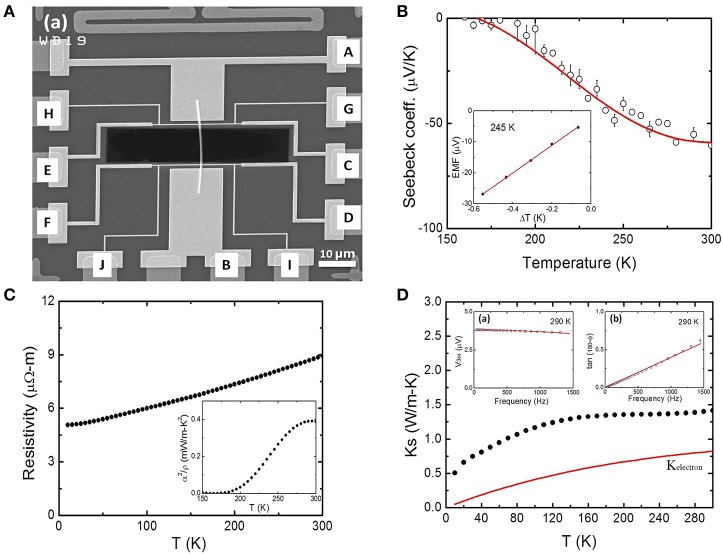
**(A)** The SEM image of the measurement platform with nanobelt suspended on the open window; **(B)** the Seebeck coefficient measurement; **(C)** the resistivity measurement, inset represents power factor for the nanobelt; **(D)** the thermal conductivity result measured for Bi1.75Sb0.25Te2.02 nanobelt with thickness 250 nm by 3*ω* method. Reproduced from Lee et al. (2013).

**Figure 3:**
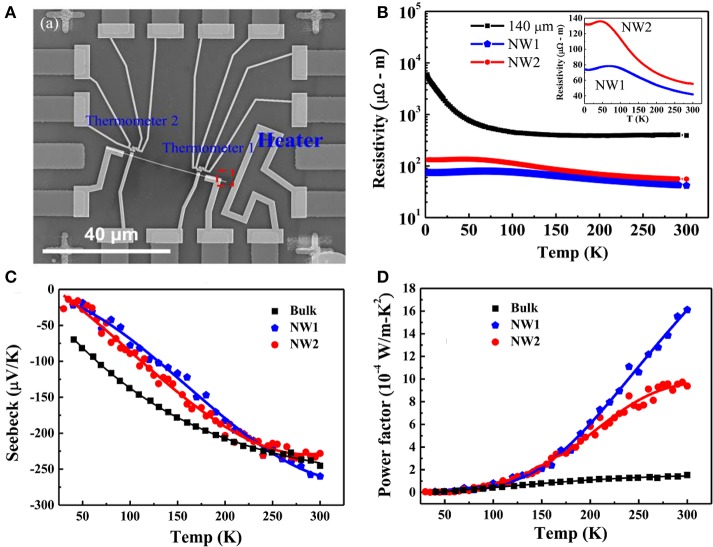
**(A)** A SEM image of the device for Seebeck coefficient and electrical conductivity measurements; **(B)** resistivity measurements; **(C)** Seebeck coefficient measurements; and **(D)** power factor for BSTS specimens of NW1 (180 nm in diameter), NW2 (230 nm in diameter), and the bulk material (thickness of 140 *μ*m). Reproduced from Hsiung et al. (2015).

Large enhancement of *ZT* inside quantum wires is predicted through theoretical studies due to its additional electron confinement. Hicks and Dresselhaus (1993) consider that nanowires can deliver higher thermoelectric performance because of stronger quantum confinement and enhanced phonon scattering, in comparison to bulk counterparts. Till now there have been various reports on the ZT enhancement in one-dimensional materials. Dedi et al. (2013) reported PbTe nanowire with diameter of 217 nm synthesized by stress induced method exhibited a maximal thermopower of −342 *μ*VK^−1^ at 375 K, which is two times larger than that of its bulk counterpart due to increase in the DOS of electrons near the Fermi level in the nanowires. The thermopower and power factor of the nanowires are shown in Figure 1. Measurement techniques for thermal conductivity of nanowires are always difficult and challenging, recently Lee et al. (2013) reported self-heating 3-omega technique that was applied to characterize the thermal conductivity of individual single crystalline Bi_1.75_Sb_0.25_Te_2.02_ nanobelt with thickness 250 nm that was prepared by On-Film Formation method. This platform provides an opportunity to measure the TE properties including structure analysis on single nanowire, which would help improve the reliability of the resulting *ZT*-value. The measurement platform, power factor and thermal conductivity of the nanowires are shown in Figure 2. Boukai et al. (2008) also reported a large enhancement in *ZT* at low temperatures (~150 K) due to phonon drag effects (heat current affecting electrical transport). This is the first time it has been claimed that phonon-drag can enhance *ZT* significantly. The argument is that in rough nanowires, the Seebeck coefficient can be increased by the transport of certain phonon modes which have minimal contribution to thermal conductivity. An interesting study published by Hsiung et al. (2015) reported *ZT* = 0.36 can be obtained at room temperature for 180 nm diameter topological insulator Bi_1.5_Sb_0.5_Te_1.7_Se_1.3_ (BSTS) nanowires synthesized by stress-induced method, representing 10 times higher than compared to its bulk counterpart because of surface-dominated transport and large insulating bulk state in the BSTS nanowires. The thermal conductivity and power factor of the nanowires are shown in Figure 3.

### Three-dimensional TE materials: Nanocomposites

Nanostructured materials are among the strongest candidates for thermoelectric applications, as they offer a route to suppressing thermal conductivity without hindering electrical properties. Second phase endotaxial nanostructuring (Ikeda et al., 2007), metal nanoparticle decoration (Lee et al., 2012), and most recently all-length scale phonon scattering (Biswas et al., 2012) have been experimentally proved to be effective routes to improve the ZT through significant reduction of the lattice thermal conductivity, *κ*_lat_.

#### AgSbTe_2_ based chalcogenide

**Figure 4:**
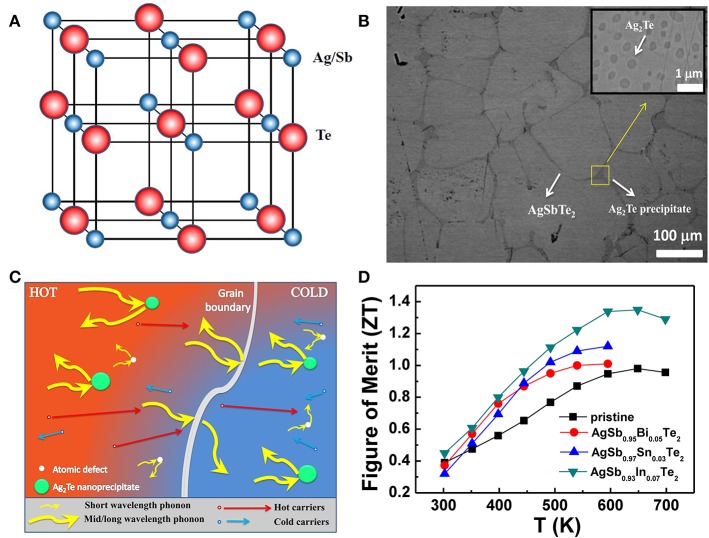
**(A)** Shows cubic rocksalt structure of AgSbTe2 compound. **(B)** Ag2Te nanoprecipitates dispersed in AgSb0.97Sn0.03Te2 matrix. Reproduced from Mohanraman et al. (2014a). **(C)** Diagrammatic representation of phonon scattering mechanisms and flow of hot and cold charge carriers inside a bulk TE material and **(D)** ZT as a function of temperature for Ag-Sb based chalcogenides as thermoelectric materials.

The best studied bulk nanocomposite material is based on p-type Silver Antimony Telluride (AgSbTe_2_), one of the traditional thermoelectric materials, which spontaneously forms nanostructures efficiently scatters phonons, without the need for artificial nanostructuring confirmed from neutron scattering and high resolution transmission electron microscope (HRTEM) investigations (Ma et al., 2013). Several studies have reported on effect of natural formation of nanoscale impurities on matrix and its contribution on ZT improvements in bulk nanostructured AgSbTe_2_ materials come from very large reduction in *κ*_lat_(Xu et al., 2010; Zhang et al., 2010; Du et al., 2011). The Ternary chalcogenide AgSbTe_2_ has already gained attention for both thermoelectric and optical phase-change applications, because of its extremely low thermal conductivity, *κ*_tot_ = 0.6 ~ 0.7 W/m/K (Hockings, 1959; Morelli et al., 2008). AgSbTe_2_ is widely identified as a rock salt NaCl type (Fm-3m) where Ag and Sb randomly occupying the Na site whereas Te is located at the Cl position shown in Figure 4A. Its lattice component *κ*_lat_, dominates largely on the total thermal conductivity, which is related to the propagation of phonons. The *κ*_lat_ in rock salt AgSbTe_2_ is about three fold lower than that of PbTe at around room temperature. It has been reported that band gap ~0.35 eV at room temperature were obtained by optical diffuse reflectance measurements, whereas strong degenerate nature reflects the electrical conductivity. Recently, the AgSbTe_2_ compound has attracted considerable attention in constructing so-called bulk nanostructured TE materials, such as (AgSbTe_2_)_1−*x*_(PbTe)_*x*_ (LAST-m) (Hsu et al., 2004), (AgSbTe_2_)_1−*x*_(GeTe)_*x*_ (TAGS) (Yang et al., 2008), and AgSbTe_2_–SnTe (Chen et al., 2012) with excellent TE properties. TAGS based alloys, which have been studied for many years and used in National Aeronautics and Space Administrative (NASA) missions since the early 1970s. The LAST-m system is an interesting bulk-grown material that spontaneously forms nanostructures during cooling from the melt.

Recent studies has been reported that element doping or substitution technique has succeeded in enhancing the thermoelectric performance of AgSbTe_2_ materials by tuning its electrical and thermal properties (Jovovic and Heremans, 2009). Moreover, the doping of appropriate semiconductor material is a potential way to enhance the thermoelectric properties of AgSbTe_2_ based alloy by reducing its lattice thermal conductivity and adjusting its carrier concentration. Du et al. (2010) investigated AgSbTe_2_ compounds by selenium (Se) doping; the electrical conductivity was enhanced greatly with an increase in the Se doping concentration. In 2014, Mohanraman et al. (2014a) reported on AgSbTe_2_ compound doped with tin (Sn), the Ag_2_Te nanoprecipitates with feature size of 100–500 nm were observed in AgSbTe_2_ matrix are effective in scattering the phonons with mid-to-long mean free paths shown in Figures 4B,C and achieved a ZT ~ 1.1 at 600 K representing an enhancement greater than 20% compared with a pristine sample shown in Figure 4D.

Moreover, recent study published by Mohanraman et al. (2013) on doping effect of bismuth (Bi) on AgSbTe_2_ material demonstrated that the Bi doping has significantly enhanced phonon scattering process through point defects over the entire temperature range, they possessed lower thermal conductivity and achieved a high *ZT*-value ~ 1.0 at 570 K shown in Figure 4D. Mohanraman et al. (2014b) reported on influence of indium (In) doping in AgSbTe_2_ material, the results showed enhanced power factor over 25–30% because of the increase in Seebeck coefficient related to decreased in carrier concentration and increase of the effective mass caused by In doping whereas lattice thermal conductivities were reduced substantially because of lattice mismatch arise from the dopants and host atoms having different atomic weights and thus resulted in enhanced phonon scattering. The highest ZT = 1.35 is achieved for Ag(Sb_0.97_In_0.03_)Te_2_ sample at 650 K shown in Figure 4D has promising applications in TE power generation in the intermediate temperature range. Furthermore, various studies on AgSbTe_2_ based alloys shows that the thermoelectric performances have been greatly improved by suitable types of dopants. Techniques such as doping or substitution have considerably decreased the lattice thermal conductivity, particularly in the high temperature range. All the results show that doping technique for enhancement of thermoelectric performance for AgSbTe_2_ based composites is reliable.

#### Bi_2_Te_3_—bulk nanocomposites

**Figure 5:**
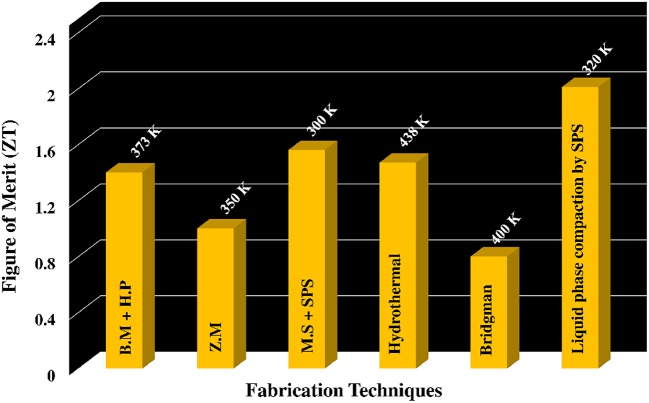
**Best ***ZT***-values obtained with the various synthesis method for Bi2Te3 based alloys**. BM, Ball Milling; HP, Hot Press; ZM, Zone Melting; MS, Melt spinning; SPS, Spark Plasma Sintering.

Bi_2_Te_3_ based alloys, the excellent TE materials at room temperature, are extensively used for the commercial thermoelectric devices for thermo-cooling application. Significant enhancement in the *ZT*-value of Bi_2_Te_3_ based bulk materials has been reported recently (Wood, 1988; Zhao et al., 2005; Cao et al., 2008; Poudel et al., 2008; Xie et al., 2009; Kim et al., 2015) shown in Figure 5. Poudel et al. (2008) reported nanostructure p-type Bi_x_Sb_2−x_Te_3_ system fabricated by mechanical milling followed by hot pressing, exhibited ZT ≈ 1.4 at 373 K. In their study reveals that ZT enhancement is partially attribute to reduction of *κ*_lat_ due to scattering at the grain boundary and the presence of nanoprecipitates. Melt spinning followed by spark plasma sintering (SPS) method fabricated bulk nanocomposite p-type (Bi,Sb)_2_Te_3_ ingot with a *ZT*-value of 1.56 at 300 K published by Xie et al. (2009). The material features nanocrystalline domains embedded in matrix composed of 5–15 nm nanocrystals with coherent grain boundaries are believed to attribute for significant reduction of thermal conductivity without degrading the electrical properties. Cao et al. (2008) obtained a high *ZT*-value of 1.47 at 438 K for Bi_2_Te_3_/Sb_2_Te_3_ bulk nanocomposite with nanoscale laminated structures prepared by a simple route involving hydrothermal synthesis and hot pressing. Kim et al. (2015) reported record *ZT*-value of 2.01 at 320 K due to generation of dislocation arrays at grain boundaries in Bi_0.5_Sb_1.5_Te_3_ by liquid phase compaction greatly reduce their thermal conduction, leading to an enhancement of their thermoelectric conversion efficiency.

In our point of view, nanocomposites and controlled two-dimensional coated nanostructures are the solution to effectively minimize thermal conductivity and promote figure of merit (ZT). Lan et al. (2012) demonstrated a method to introduce nano-coating structures into surface of bulk material by using a hydrothermal process. A fine crystalline layer of Bi_2_Te_3_ was coated onto the surfaces of Bi seed micron-sized particles. After that, highly densified pellets were successfully obtained by the subsequent hot-press at around 400 K and a uniaxial pressure of 680 MPa for 30 min. Binary-phase particles composed of micro-sized Bi particles and nanosize Bi_2_Te_3_ were prepared via the aforementioned process.

**Figure 6:**
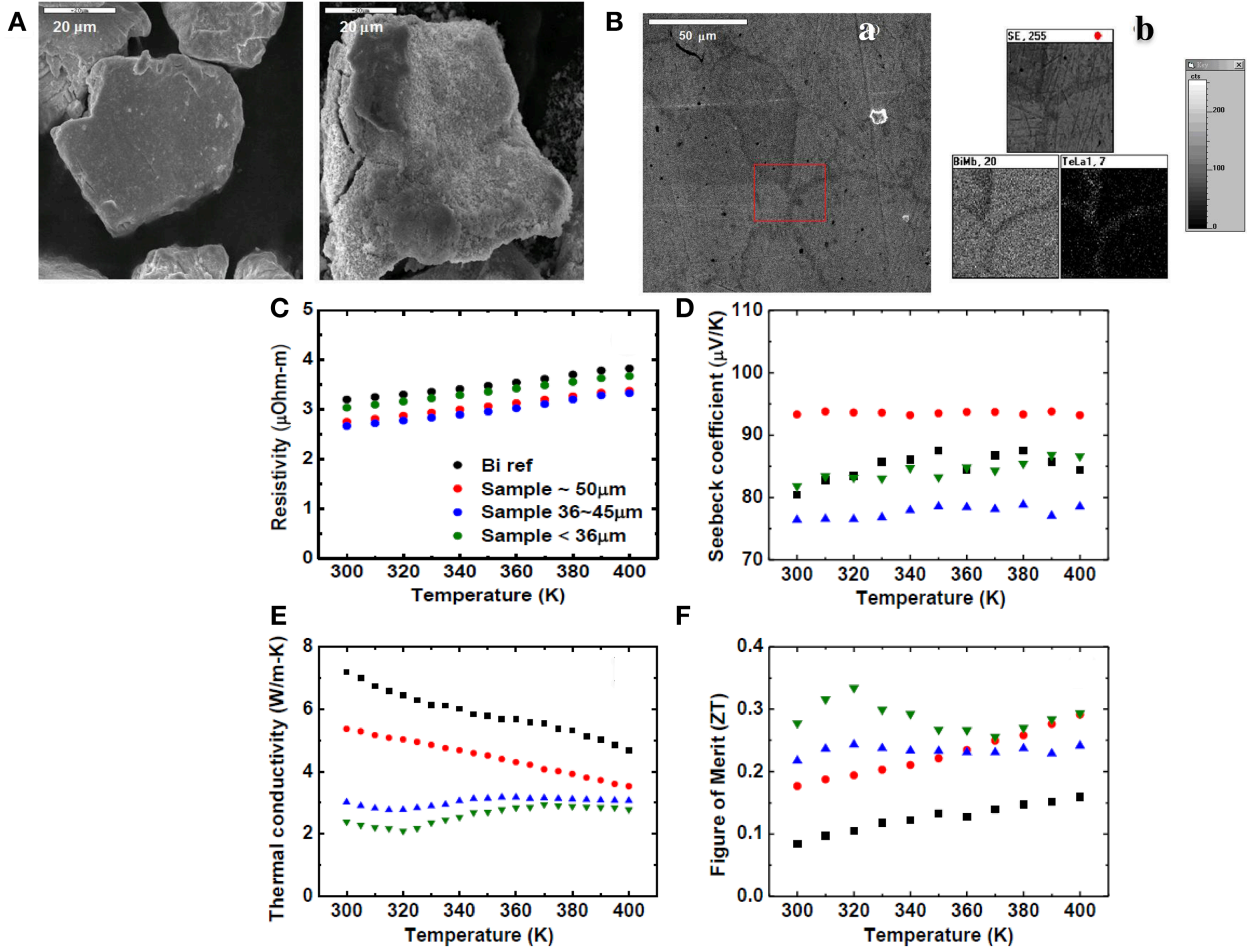
**(A)** SEM images of the particles before (left) and after (right) hydrothermal coating process. **(B) a**. Shows SEM micrograph of bulk after grinding. **b**. EDS mapping analysis shows the amounts of Te were more along the boundary which is clearly indicate the existence of a thin Bi2Te3 layer. A plot of the **(C)** the electrical resistivity, **(D)** the Seebeck coefficient **(E)** the thermal conductivity and **(F)** the TE figure of merit (ZT) vs. temperature for hot pressed samples with various sizes of seed particles and a crystalline Bi ingot. Reproduced from Lan et al. (2012).

The morphology of the samples is studied by SEM. In Figure 6A, the difference between before and after the coating process is shown. The surface of particles after the hydrothermal process is fuzzier and the EDS analysis shows larger amounts of Te on the boundary of the two particles. This indicates that Bi_2_Te_3_is very small, or a very thin layer of Bi_2_Te_3_ is coated on the surface of Bi grains. Figure 6B shows that the existence of boundaries in the bulk sample is more obvious after grinding. Figure 6Ba shows that grain boundaries can still be observed by SEM. The EDS mapping analysis (Figure 6Bb) shows larger amounts of Te along the boundary, and that the coating layer is less than few micrometers.

The nano-coating process even slightly decreases electrical resistivity and maintains the values of Seebeck coefficient at the same value shown in Figures 6C,D respectively. Furthermore, when the sizes of Bi seed particles decrease, the total thermal conductivity decrease with the same trend is shown in Figure 6E. All four samples show similar downward tendency. Because the TE property of Bi_2_Te_3_ near the room temperature is at an advantageous position, the small decrease of electrical resistivity and the values of Seebeck coefficient can be explained by the existence of Bi_2_Te_3_ thin layer. Furthermore, the thermal conductivity *κ* was gradually diminished while the grain size was reduced. This also indicates that the quantity of grain boundaries is influenced by the thermoelectric property. The coating process might scatter the phonons but not the electrons. Moreover, the electrical resistivity was also diminished when the Bi_2_Te_3_ was coated on. This may be because the coating layer is more flexible than the core material making for better contact of particles than with the single phased material. Hence, the nano-coating process can increase the figure of merit (ZT) because the coating layers provide more boundaries and also prohibit the aggregation of particles within the sintering process. Increasing the number of grain boundaries could efficiently reduce the thermal conductivity without the reduction of electrical conductivity. We found that reducing thermal conductivity leads to a dimensionless figure of merit ZT ~ 0.278 at ~300 K shown in Figure 6F, more than an appreciable improvement over commercial Bi powder treated with the same hot-press process. We propose a new route for developing high performance Bi nano-composites by using a hydrothermal nano-coating process, which have even broader prospects for commercial applications. The combination of nano-coating layers and Bi seed particles with subsequent hot-pressed process affect the TE properties of the Bi, leading to a significant enhancement of the figure of merit. The enhancement of ZT was primarily influenced by an appreciable reduction in the thermal conductivity. It was due to presence of nanostructured regions existing within the material as the result of our processing route.

#### PbTe nanocomposites

**Figure 7:**
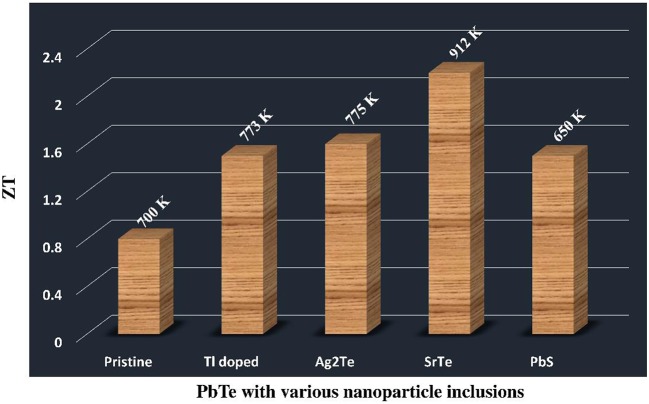
**Current state of the art in PbTe nanocomposites; the TE figure of merit ZT with various nanoparticle inclusions**.

PbTe alloys are one of the premiere TE materials for intermediate range temperature (500–800 K) applications and played a key role in radioisotope thermoelectric generator for deep space exploration program as a power source. Recently, Kanatzidis group has published many reports on significant improvements in the thermoelectric properties of PbTe based alloys by nanostucturing and also modification in density of states through band structures (Zhao et al., 2014) shown in Figure 7. A recent study published by Biswas et al. (2012) has reported a high record of *ZT*-value of 2.2 at 915 K for p type PbTe-SrTe system via grain boundary phonon scattering enabled by nanostructuring to reduce the thermal conductivity.

#### CoSb_3_ nanocomposites

**Figure 8:**
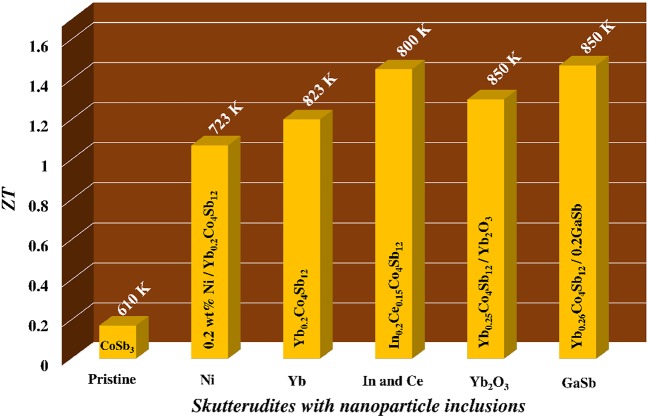
**Summary of some of the best ***ZT***-values obtained with the various nanoparticle inclusions for CoSb3 skutterudites**.

CoSb_3_ based skutterudites are highly promising candidate for medium temperature TE power generation applications because both n and p type materials with high performance can be obtained in the same material system. The most remarkable feature of this compound is that the cage like open structure are found and can be filled with foreign atoms acting as phonon rattlers which scatter phonons strongly thus drastically reduces the thermal conductivity. Nanocomposites, as an efficient way to reduce the thermal conductivity via grain boundaries and nanoinclusions, have also been used in CoSb_3_ based TE materials. Significant advances have been made in recent years with various kinds of nanoinclusion filling the cages in this compound (Bertini et al., 2003; Zhao et al., 2006; Li et al., 2009; Yang et al., 2009; Xiong et al., 2010; Fu et al., 2015) and shown in Figure 8. Fu et al. (2015) reported formation of core-shell microstructure in compounds doped with 2% of Ni, has enhanced ZT to 1.07 at 723 K. Li et al. (2009) observed *in situ* formation of InSb nanoislands in the In_0.2_Ce_0.5_Co_4_Sb_12_ nanocomposite with enhanced ZT up to 1.43 at 800 K by significant reduction in thermal conductivity. Zhao et al. (2006) fabricated Yb_0.25_Co_4_Sb_12_ nanocomposite and well distributed Yb_2_O_3_ particles synthesized by *in situ* reaction. The Yb_2_O_3_ nanoinclusions located at the grain boundaries are effective in scattering phonons, there by increases the figure of merit and achieved peak ZT of 1.3 at 850 K. Xiong et al. (2010) has reported formation of GaSb nanoinclusions for the (GaSb)_0.2_-Yb_0.26_Co_4_Sb_12_ nanocomposite exhibiting peak *ZT*-value of 1.45 at 850 K.

## Conclusions and outlook

This review summarizes the recent progress of nanowires; Ag-Sb based alloys, PbTe, CoSb_3_ skutterudites, and Bi_2_Te_3_ based nanocomposites. Nanostructures such as nanoprecipitates, controlled two-dimensional coated nanostructures, nanoinclusion to atomic defects, and nanoscale inhomogeneities have been found to be potential routes for reducing thermal conductivity to a greater extend without hindering much on electrical conductivity, resulting in an enhanced figure of merit for the bulk nanocomposite material. However, additional approaches such as carrier-energy filtering or quantum confinement effects will likely be key role for enhancing power factor to achieve further significant *ZT* enhancement. Overall from our practical point of view, bulk nanocomposites shows more exciting than nanowires or nanobelts because the former can reduce the expenses, scale-up, and thermal management issues normally related with the later.

### Conflict of interest statement

The authors declare that the research was conducted in the absence of any commercial or financial relationships that could be construed as a potential conflict of interest.
